# Precise Protein Photolithography (P^3^): High Performance Biopatterning Using Silk Fibroin Light Chain as the Resist

**DOI:** 10.1002/advs.201700191

**Published:** 2017-07-06

**Authors:** Wanpeng Liu, Zhitao Zhou, Shaoqing Zhang, Zhifeng Shi, Justin Tabarini, Woonsoo Lee, Yeshun Zhang, S. N. Gilbert Corder, Xinxin Li, Fei Dong, Liang Cheng, Mengkun Liu, David L. Kaplan, Fiorenzo G. Omenetto, Guozheng Zhang, Ying Mao, Tiger H. Tao

**Affiliations:** ^1^ Department of Mechanical Engineering The University of Texas at Austin Austin TX 78712 USA; ^2^ State Key Laboratory of Transducer Technology Shanghai Institute of Microsystem and Information Technology Chinese Academy of Sciences Shanghai 200050 China; ^3^ School of Graduate Study University of Chinese Sciences of Academy Beijing 100049 China; ^4^ Department of Neurosurgery Huashan Hospital of Fudan University Wulumuqi Zhong Road 12 Shanghai 200040 China; ^5^ Jiangsu University of Science and Technology No. 2 Mengxi Road Zhenjiang Jiangsu 212003 China; ^6^ Department of Physics and Astronomy Stony Brook University Stony Brook NY 11794 USA; ^7^ School of Physical Science and Technology ShanghaiTech University Shanghai 200031 China; ^8^ Department of Biomedical Engineering Tufts University Medford 02155 USA

**Keywords:** biopatterning, protein photolithography, silk fibroin light chain

## Abstract

Precise patterning of biomaterials has widespread applications, including drug release, degradable implants, tissue engineering, and regenerative medicine. Patterning of protein‐based microstructures using UV‐photolithography has been demonstrated using protein as the resist material. The Achilles heel of existing protein‐based biophotoresists is the inevitable wide molecular weight distribution during the protein extraction/regeneration process, hindering their practical uses in the semiconductor industry where reliability and repeatability are paramount. A wafer‐scale high resolution patterning of bio‐microstructures using well‐defined silk fibroin light chain as the resist material is presented showing unprecedent performances. The lithographic and etching performance of silk fibroin light chain resists are evaluated systematically and the underlying mechanisms are thoroughly discussed. The micropatterned silk structures are tested as cellular substrates for the successful spatial guidance of fetal neural stems cells seeded on the patterned substrates. The enhanced patterning resolution, the improved etch resistance, and the inherent biocompatibility of such protein‐based photoresist provide new opportunities in fabricating large scale biocompatible functional microstructures.

## 1 Introduction

Precise patterning of micro and nanostructures using polymer‐based biomaterials has widespread applications including drug release, degradable implants, tissue engineering, and regenerative medicine.^1^, ^2^, ^3^, ^4^ In this context, natural silk proteins obtained from cocoons of the silkworm *Bombyx mori* provide “green” alternatives to synthetic materials with advantages such as superior mechanical properties (strength and toughness), outstanding biocompatibility and biodegradability, and controllable water‐solubility and degradation rate.^1^, ^5^, ^6^, ^7^

Natural silk fibers from *B. mori* cocoons exist in a self‐assembled fibrous configuration, in which a mechanically robust protein–fibroin (≈75%, w/w) comprises the core, surrounded by a glue protein–sericin (≈25%, w/w).^8^ To date, several techniques, such as Electron beam (E‐beam) writing,^9^, ^10^ imprinting,^11^, ^12^ molding,^13^, ^14^ electrospinning,^15^ embossing,^16^, ^17^ inkjet printing,^18^ and photolithography (P^3^)^19^, ^20^ have enabled the development of a variety of material formats, including hydrogels, fibers, particles, and films using fibroin or sericin as well as their blends with other materials.^3^, ^21^, ^22^ Photolithography, in particular, remains one of the most appealing techniques for scalable biomanufacturing as it is Complementary Metal Oxice Semiconductor (CMOS)‐compatible and can rapidly fabricate high fidelity micro/nanopatterns in parallel—in contrast, scanning‐probe lithography and electron beam lithography for biomanufacturing use serial manufacturing techniques.^23^, ^24^, ^25^, ^26^, ^27^

Patterning of silk microstructures using UV‐P^3^ has been successfully demonstrated where either silk fibroin or sericin was chemically modified to be photoreactive and then served as the photoresist.^28^, ^29^ Cell culture studies have been conducted to verify the biocompatibility of silk protein resists after the chemical modification and lithographic process.^28^, ^29^ Though very promising, compared to their commercial counterparts based on synthetic polymers, current silk protein resists still suffer from issues such as relatively low resolution and pattern contrast in terms of lithographic patterns, mainly due to the inevitable wide molecular weight distribution (ranging from a few tens to a few hundreds of kDa for both silk fibroin and sericin proteins) during the degumming process for protein extraction.^30^ Such limits hinder their practical use in precision biopatterning and the semiconductor industry where reliability and repeatability are paramount. Proteins with more uniform molecular structures (such as well‐defined chain lengths and molecular weights) and preferably more active group sites for further functionalization have yet to be explored for high‐performance protein‐based photolithography.

In this study, we report on a precise protein photolithography for high‐performance biopatterning using the well‐defined silk fibroin light chain as the basic resist material. Silk fibroin is mainly composed of two components, namely heavy chain (H‐fibroin, ≈85%, w/w) and light chain (l‐fibroin, ≈15%, w/w), which are linked by a single disulfide bond between Cys‐c20 of H‐fibroin and Cys‐172 of l‐fibroin.^31^, ^32^, ^33^ Compared to silk fibroin and sericin proteins, l‐fibroin has a well‐defined molecular weight of ≈26 kDa.^34^ It also has a higher proportion of undifferentiated and hydrophilic amino acid composition than H‐fibroin, which facilitates facile chemical modification for the synthesis of a variety of biologically and chemically functional photoresists.^35^, ^36^, ^37^

## 2 Results and Discussion

**Figure 1:**
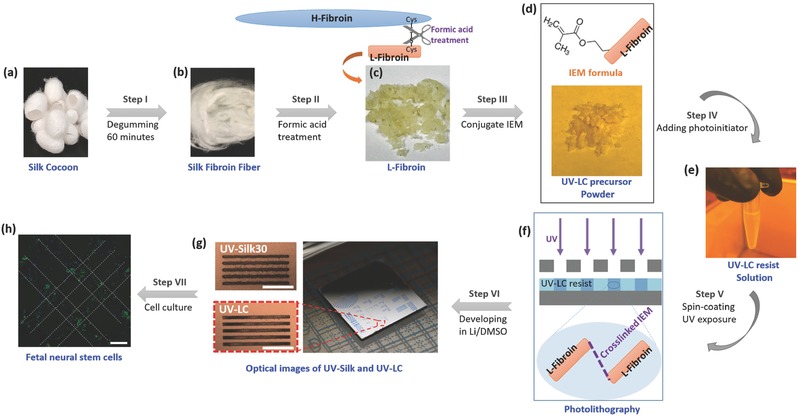
Synthesis of the UV‐reactive silk l‐fibroin (UV–LC) and the result of photolithography using UV–LC as a negative resist. a) *B. mori* cocoons are degummed for 60 min to obtain b) silk fibroin, and c) the l‐fibroin is then separated from the silk fibroin using formic acid; d) photoactive l‐fibroin (UV–LC precursor) is obtained by conjugating IEM to the l‐fibroin; e) by adding the photoinitiator (Irgacure 2959), the UV–LC resist can be synthesized; f) photolithography using UV–LC resist; g) optical images of the fabricated patterns (linewidth: 5 µm, zoom‐in image) shows that UV–LC has better lithographic performance than UV–Silk30. Scale bar: 50 µm. h) Dark‐field stereomicroscopic photograph of double immunofluorescence staining with Nestin (green fluorescence) and nuclear staining (blue DAPI staining) of fetal neural stems cells that were guided to be cultured on a micropatterned UV–LC resist (dash line) on a silicon substrate. Scale bar: 100 µm.

**Figure** **1** illustrates the material synthesis, functionalization, and photolithographic results of UV‐reactive silk l‐fibroin (UV–LC) resists. The *B. mori* silkworm cocoons were first cut into small pieces and degummed for 60 min to remove sericin using a previously reported process^38^ (Figure 1a,b). Formic acid was used to break the covalent disulfide bonds between H‐fibroin and l‐fibroin, and to separate silk fragments based on their different solubilities in formic acid without causing severe protein degradation.^39^, ^40^ The soluble fractions (i.e., l‐fibroin) were harvested and air‐dried (Figure 1c). The l‐fibroin was modified to be photoreactive by conjugating a photoreactive reagent of 2‐isocyanatoethyl methacrylate (IEM) to l‐fibroin's side groups, yielding a photocrosslinkable UV–LC precursor (Figure 1d). The UV–LC precursor was then dissolved in 1,1,1,3,3,3‐hexafluoro‐2‐propanol (HFIP, Sigma Aldrich, St. Louis, MO). An organic photoinitiator of Irgacure 2959 (Sigma Aldrich, St. Louis, MO) was added 0.5% (w/v) into the UV–LC precursor solution to generate (and transfer) reactive species (free radicals in this case) when exposed to UV radiation (Figure 1e). The UV–LC resist solution (2%, w/v) was spin coated on a silicon or glass substrate to form a resist layer with a controllable thickness ranging from 50 nm to several micrometers which was then exposed through a photomask (Figure 1f). In this case, the UV–LC resist acted as a negative photoresist which can be crosslinked due to IEM in the presence of UV light (followed by the development step) to generate wafer‐scale micropatterns on silicon and glass substrates via standard UV photolithography (Figure 1g). UV–LC microstructures were tested as cellular substrates and for the spatial guidance of fetal neural stems cells which were seeded on micropatterned surfaces and incubated for 3 d. Cells tended to preferentially attach to the UV–LC protein patterns in comparison to the surrounding surface (i.e., silicon in this case) (Figure 1h, more details in Figure 5, also see Supporting Information). Note that the sensitivity of UV–LC resists can be readily tuned by regulating the IEM molecules conjugated into l‐fibroin. Additionally, the presence of unmodified amino acids can enable further functions (e.g., association with favorable cellular interactions and the production of multifunctional biomaterial architectures) via concurrent or subsequent modification strategies.^41^ In this study, the IEM molecules were intentionally designed to exceed the population of available amino acids conversion to fully occupy nearly all active group sites on the protein chains to better investigate the underlying mechanism of photo‐only‐induced formation of crosslinked silk micro/nanostructures.

**Figure 2:**
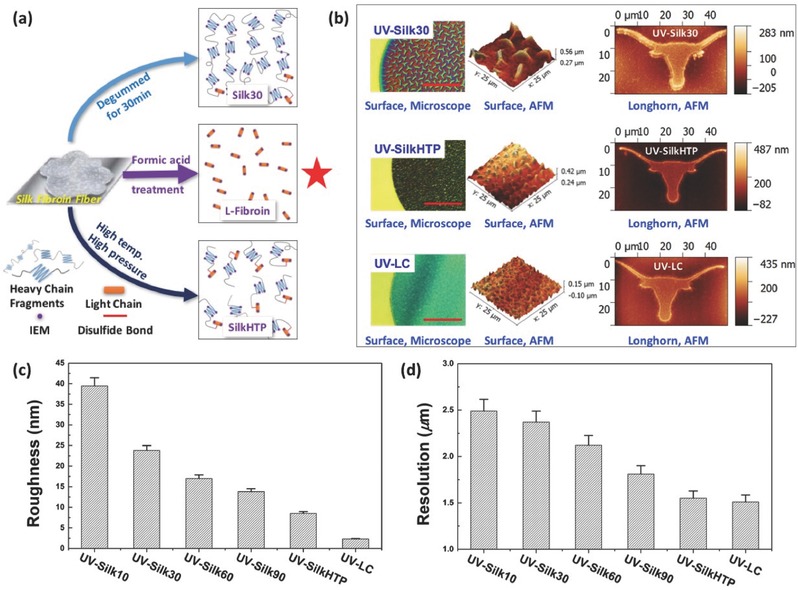
Characterization and analysis of patterns fabricated by protein photolithography using different types of silk‐based materials (e.g., UV–Silk30, UV–SilkHTP, and UV–LC). a) Schematic comparison between structures of UV–LC and UV–Silk (including both UV–Silk30 and UV–SilkHTP) precursor, where UV–Silk30 has longer protein chains than UV–HTP. UV–LC contains only l‐fibroin; b) morphological characterization (using an optical microscope and AFM, scale bar: 200 µm) of micropatterns fabricated by protein photolithography using UV–Silk30, UV–SilkHTP, and UV–LC. It shows that the UV–LC can achieve better resolution and surface smoothness than UV–Silk30 and UV–SilkHTP; c,d) quantitative analysis of resolution and surface roughness of micropatterns fabricated using various UV–Silk and UV–LC. The result is consistent with the observations from optical and AFM images.

A variety of photoreactive fibroin (UV–Silk) resists with varied degumming conditions (thus varied protein chain lengths and molecular weight distributions) have been prepared for comparison using a previously reported method.^29^ Note that UV–Silk resists consist of both H‐fibroin and l‐fibroin fragments, while UV–LC only has l‐fibroin. **Figure** **2**a schematically shows the (simplified) molecular structures of some example UV–Silk and UV–LC precursors, including UV–Silk made of silk fibroin fibers degummed for 30 min (UV–Silk30), degummed at high temperature (121 °C) and pressure (25 psi) for 4 h (UV–Silk high temperature and pressure [HTP]), and l‐fibroin protein, respectively. In general, longer degumming time results in shorter but more uniform silk fibroin fragments. Therefore, compared to UV–Silk30, the H‐fibroin fragments in UV–SilkHTP are generally shorter but more uniform due to the high temperature and pressure treatment conditions during its extended degumming process.^42^ In comparison, UV–LC provides a promising route serving as the basic molecular blocks for precise protein photolithography thanks to its well‐defined and evenly distributed protein chains.

The surface morphology and fidelity of as‐fabricated micropatterns on isopropyl alcohol (IPA) cleaned silicon substrates using UV–LC and UV–Silk protein resists were characterized and compared using atomic force microscopy (AFM) and an optical microscope, showing that lithographic performances including the spatial resolution, pattern sharpness, and surface morphology/roughness strongly depend on the molecular structures of as‐used protein resists (Figure 2b). AFM results show that UV–LC has the best surface roughness with a root mean square roughness of ≈2.3 nm while UV–SilkHTP (≈8.5 nm) is better than UV–Silk30 (≈23.8 nm), over an area of 5 × 5 µm. We postulate that, during drying, silk fibroin proteins spontaneously form micro and nanoscale wrinkled patterns guided by a diffusion‐limited aggregation process^43^ which has been observed in the assembly of a range of materials including colloids, polymer thin films,^44^ peptides,^45^, ^46^ and proteins.^47^ This is partially due to the polarity mismatch between photoreactive silk resists consisting of strongly polar side groups, such as hydroxyl, carboxyl, and amino groups (thus strongly polar) and the IPA‐treated silicon (weakly polar) substrate, which can be improved by appropriate surface treatment of silicon substrates (details in the Supporting Information). The mismatch increases with the protein chain length and uneven distribution of the molecule weight where the internal molecular polarity difference within the protein chain becomes more predominant due to increased ratio between the hydrophobic H‐Fibroin fragments and the hydrophilic l‐Fibroin fragments (Figure 2c).

Figure 2d shows that, under the same lithographic conditions (i.e., exposure duration and development time), the resolution (which was determined by the minimum distinguishable feature size in our case, see the Supporting Information) of UV–Silk resists improves with the increasing degumming time, due to the decreased (and more uniformly distributed) molecular weight. The UV–SilkHTP and UV–LC provide better lithographic performances in terms of resolution, yielding minimum feature sizes of 1.54 and 1.51 µm, respectively, close to the minimum designed feature size of the mask (1.5 µm), which was chosen based on the capabilities of our current photolithography setup.

**Figure 3:**
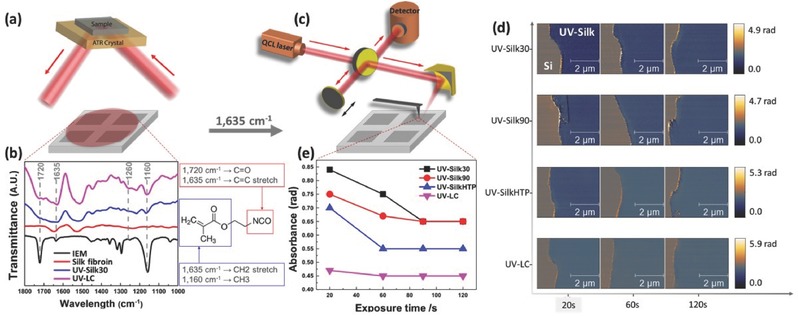
Structural characterization of the UV–Silk and UV–LC using FTIR and s‐SNOM. a) Schematic of ATR–FTIR setup, where the sample is illuminated from the back of the ATR crystal; b) FTIR spectrum of IEM, silk fibroin protein, UV–Silk, and UV–LC. The peaks vanish in the UV–Silk and UV–LC, indicating the binding of IEM on silk fibroin and l‐fibroin; c) schematic of the s‐SNOM system. An infrared laser is focused onto the AFM tip, and the scattered signal is collected by the detector; d,e) IR nanoimaging and absorbance (acquired by s‐SNOM measurement performed at 1635 cm^−1^) of UV–Silk30, UV–Silk90, UV–SilkHTP, and UV–LC with various exposure time. The disappearance of the absorbance with increasing exposure time indicates the increasing crosslinking degree of IEM until about 90 s, after which time all the available IEM active conjugated acrylate group sites are crosslinked.

The underlying crosslinking mechanism via the conjugation of the multifunctional acrylate moiety (i.e., IEM in this case) to silk fibroin proteins (including both H‐fibroin and l‐fibroin) and l‐fibroin only—at macro and nanoscale—was investigated via both Fourier transform infrared spectroscopy (FTIR) and the scattering‐type scanning near‐field optical microscopy (s‐SNOM), respectively. Three characteristic peaks (red curve, **Figure** **3**b) were found in Amide I (1600–1700 cm^−1^), Amide II (1500–1600 cm^−1^), and Amide III (1200–1300 cm^−1^) bands for bulk silk fibroin proteins measured by FTIR in the attenuated total reflection (ATR) mode with an aperture size of several tens of micrometers (Figure 3a), which gradually decreased after the introduction of the photoactive component IEM (Figure 3b). Three prominent peaks (blue and pink curves for UV–Silk and UV–LC, respectively) surged at 1720 cm^−1^ (C=O stretch), 1635 cm^−1^ (terminal C=C stretch), and 1160 cm^−1^ (C—O stretch), which overlapped with the characteristic peaks of pure IEM.

s‐SNOM was employed to provide direct imaging and chemical identification of the thin protein layers at the nanoscale, to understand the variation of local chemical composites during crosslinking under UV exposure, and to overcome the special resolution and thickness limits of FTIR spectroscopic study.^48^ In this work, s‐SNOM (NeaSNOM, Neaspec GmbH, Germany) has been utilized for high‐resolution optical images and spectroscopic information to map out the chemical and mechanical properties of protein patterns at the nanoscale with a spatial resolution of ≈20 nm. In our setup, s‐SNOM is coupled to a tunable IR quantum cascade laser (QCL, Daylight Solutions Inc., USA) covering the broad IR spectra of the Amide I and II bands over the range from 1450 to 1750 cm^−1^ (Figure 3c, also see the Supporting Information). The near‐field phase spectrum resembles the molecular absorbance band, while the near‐field amplitude spectrum acquires a dispersive line shape similar to a far‐field reflectivity spectrum.^49^

The crosslinking degree was measured by using absorbance phase images, where the absorbance intensity at characteristic peak of crosslinking is inversely proportional to the degree of crosslink. Absorbance phase images of UV–Silk30, UV–Silk90, UV–SilkHTP, and UV–LC patterns on a silicon substrate were captured at 1635 cm^−1^, which corresponds to IEM‐induced photocrosslinking from terminal C=C group sites. As shown in Figure 3d, at 1635 cm^−1^, the phase image exhibited a strong contrast between silk and silicon (silicon is used as the reference for IR imaging) in UV–Silk30 micropatterns that were exposed for 20 s. The contrast gradually weakened with the longer exposure time indicating an increased crosslinking degree. IEM‐induced crosslinking degrees within various protein micropatterns were obtained and evaluated quantitatively (Figure 3e). The absorbance intensity of UV–Silk30 samples decreased monotonically with increasing exposure time until 90 s, indicating an increase in crosslinking degree due to IEM. The crosslinking was found to be saturated after 90 s exposure which expended all available active conjugated acrylate group sites and remained nearly constant thereafter. It is found that UV–Silk resists with lower molecular weights are easier to crosslink partially due to their higher degrees of molecular mobility and more uniform protein chain lengths. Compared to UV–Silk resists, UV–LC resist shows considerably higher sensitivity thanks to its shorter protein chain length and more available IEM side groups.

**Figure 4:**
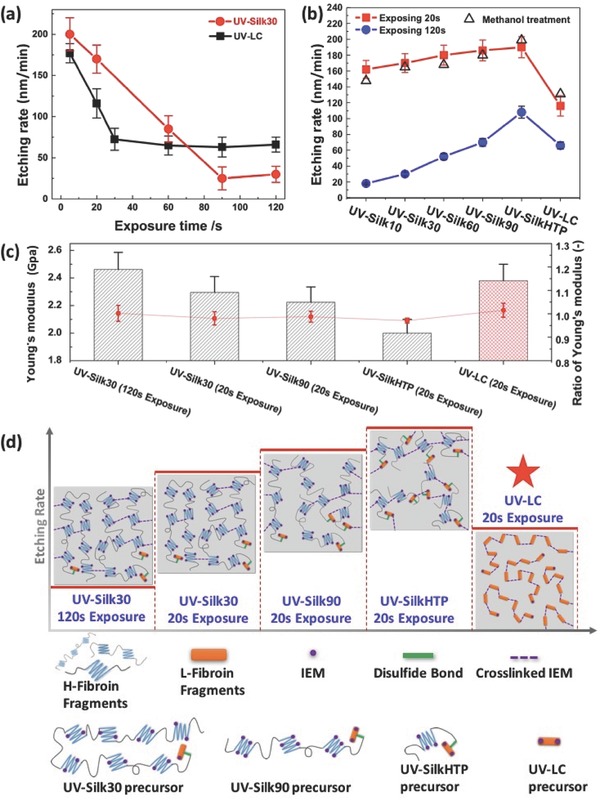
Etching rate measurements and schematic structures of various UV–Silk and UV–LC. a) Etching rate measurement of the UV–Silk30 and UV–LC with increasing exposure time. The etching rate of UV–LC decreases faster than the UV–Silk30 with increasing exposure time but reaches a constant rate that is higher than UV–Silk30; b) etching rate comparison between various UV–Silk (including both UV–Silk and methanol treated UV–Silk) and UV–LC at two exposure times (20 and 120 s). All the samples with 20 s exposure times have larger etching rate than the samples with 120 s exposure times. UV–Silk has an increasing etching rate with increasing degumming time because the mechanical strength is better with longer chain length. UV–LC has slightly less etching rate because its highly defined molecular structure help form better crystalline structure; c) Young's modulus of UV–Silk and UV–LC and ratio of Young's modulus before and after methanol treatment. It shows the similar trend with the data of etching speed, where the largest Young's modulus value corresponds to slower etching rate. It also shows no obvious change on Young's modulus by treating with methanol, suggesting no formation of beta sheet structures in UV–Silk and UV–LC resists induced by the methanol treatment; d) schematic structure and the corresponding etching rate of the photocrosslinked UV–Silk and UV–LC with 20 s exposure time and UV–Silk30 exposed for 120 s. For UV–Silk30, the etching rate decreases with longer exposure time because of the increased crosslinking degree. With the same exposure time, the etching rate increases with increasing degumming time because of the shorter chain length, and thus less mechanical strength. With 20 s exposure (partially crosslinking) UV–LC has less etching speed because its highly defined molecular structure helps it form better IEM‐induced crystalline structure.

One main use of photolithography is to pattern a resist layer which can serve as a temporary mask when etching an underlying layer. Therefore, a systematic study on the use of UV–Silk and UV–LC resists as the etching mask for pattern transfer was conducted. We have found that there are at least three factors that play an important and synergistic role in the etching performance (i.e., etching resistance) of the silk‐based microstructures, namely, (1) the average molecular weight (i.e., average protein chain length, which is determined by the degumming process (for UV–Silk resists) and protein separation process (for UV–LC resist)); (2) the photoinduced crosslinking due to IEM; and (3) the crosslinking due to the formation of beta sheets. We first investigated the dependence of etching performance on the photoinduced crosslinking within the protein matrix and the protein chain lengths. As shown in **Figure** **4**a, the etching rates of both UV–Silk30 and UV–LC resists decreased monotonically with the increased exposure time (and thus the increased crosslinking degree before saturation) and reached plateaus at ≈25.4 and ≈72.4 nm min^−1^ after UV exposure of 90 and 30 s, respectively. The etching resistance of UV–LC was initially better than UV–Silk30 since the crosslinking degree in UV–LC was considerably higher than UV–Silk30 under the same exposure conditions. With the increased exposure time, the molecular weight of silk protein chains became to play a more important role and UV–Silk30 showed a better etching resistance when both resists were fully crosslinked.

We designed two sets of experiments to systematically investigate the etching performance among a variety of silk resists (including both UV–Silk and UV–LC ones) that were (1) partially crosslinked (for 20 s exposure so that all resists were “underexposed”) and (2) fully crosslinked (for 120 s exposure so that all resists were “overexposed”) (Figure 4b). It has been found that, under same exposure conditions (for both partially and fully crosslinking cases), the etching rate of UV–Silk resists increased with the degumming time. This is mainly due to the reduced protein chain length during the prolonged degumming process which weakens the mechanical strength of the as‐prepared protein resist and causes the increase in the etching rate (Figure 4c). A schematic illustration of the underlying mechanism is given in Figure 4d. We then compared the etching performances of UV–LC resist to UV–Silk ones. In the partially crosslinking case, the UV–LC resist showed the best etching performance due to its significantly higher crosslinking degree than all UV–Silk resists (also see Figure 3e). However, for the fully crosslinking case, a competing mechanism becomes more noticeable between the crosslinking degree and the molecular weight on the etching performance. Generally, UV–LC resist has much lower molecular weight but higher crosslinking degree than UV–Silk resists under same exposure conditions. Therefore, when fully crosslinked, UV–Silk resists with relatively short degummed time showed better etching resistance than UV–LC, due to their much higher molecular weights and better mechanical strengths. UV–Silk90 shows a comparable etching resistance to UV–LC as it has higher molecular weight but less crosslinking degree. UV–SilkHTP shows the highest etching rate as it has much lower average molecular weight than other UV–Silk counterparts due to the excessive degumming time under high temperature and pressure.

Furthermore, we have found that the influence of the secondary structure of beta sheets within silk resists on their etching performances is considerably minor compared to the other two factors, namely, the average molecular weight and IEM‐induced crosslink. It is well known that methanol treatment can promote the formation of beta sheets within the silk protein matrix.^6^ No noticeable variation was found in terms of the etching resistance or Young's modulus before and after the methanol treatment for all silk resist samples (Figure 4b,c). We attribute this to the fact that the degree of IEM substitution was designed to exceed the population of amino acids conversion so to occupy almost all the active group sites on the protein chains, which hindered the formation of betasheet structures in the protein resist matrix.

**Figure 5:**
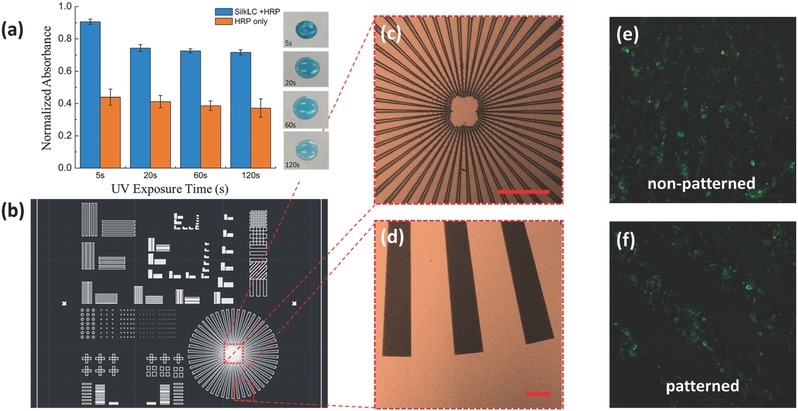
a) Bioactivity evaluation of HRP‐doped silk resist and HRP enzyme after UV exposure. The ELISA test shows that enzyme activities are negatively affected during UV exposure (as shown in the photos on the right) and silk resists can help to stabilize the bioactivities to some extent during UV exposure; b) portion of the designed photomask. c–f) Double immunofluorescence staining with Nestin (green fluorescence) and nuclear staining (blue DAPI staining) of fetal neural stem cells cultured on UC–LC substrates showing the spatial guidance of cell seeding. Scale bar: 100 µm.

One of the most compelling attributes of silk materials is their abilities to allow for the incorporation of functional elements such as labile biological components with retention of bioactivity to generate functional material formats.^50^ The effectiveness of UV–LC photolithography to large scale reproduce microscale geometries and topologies allows for functional components to be generated from silk. We therefore explore the doping and stabilization of UV–LC patterns with an enzyme of horseradish peroxidase (HRP) and the effects of the UV–LC photolithography process on its bioactivities as proof‐of‐principle demonstrations. As shown in **Figure** **5**a, the enzymatic activity of the HRP‐doped UV–LC resist was quantitatively assessed by a colorimetric enzyme‐linked immunosorbent assay (ELISA) for HRP/3,3′,5,5′‐tetramethylbenzidine (TMB) after UV exposure. The bioactivity test shows that as‐prepared patterns possess bioactivity of embedded biological molecules to some extent (i.e., HRP‐dope UV patterns turn blue after exposure to TMB) during UV–LC photolithography. Finally, UV–LC microstructures were fabricated and examined using a standard immunofluorescence assay as biocompatible cellular substrates (Figure 5b–d). Fetal neural stem cells were seeded on nonpatterned (i.e., a uniform coating of UV–LC resist w/o UV exposure) and patterned surfaces using UV–LC photolithography and incubated for 3 d. As shown in Figure 5e,f, cells were well anchored to the UV–LC substrates in both cases and tended to preferentially attach to UV–LC patterned compared to the surrounding surface (i.e., silicon in this case), showing that UV–LC micropatterns have good biocompatibility and can be used for precisely spatial cell guidance.

## 3 Conclusion

In conclusion, we report on a precise protein P^3^ for wafer‐scale, high‐performance biopatterning using chemically modified well‐defined silk l‐fibroins as the photoresist material. The lithographic and etching performance of UV–LC and UV–Silk resists have been evaluated systematically and the underlying mechanisms have been thoroughly discussed. A general guidance on the synthesis and the use of silk l‐fibroin resist has been provided. The inherent biocompatibility and the enhanced patterning resolution along with the improved surface roughness and etching performance of such protein‐based resists offer new opportunities in fabricating large‐scale high‐precision biocompatible functional micro/nanostructures.

## 4 Experimental Section

*Synthesis and Purification of Light Chain Proteins*: 60 min degummed silk fiber was weighed and dispersed in 98–100% formic acid at a range of concentrations (0.01–8%, w/v) for 30 min. The mixture was then centrifuged at 4000 rpm for half an hour to sediment the undissolved material. The supernatant was filtered using glass fiber filters to remove any remaining suspended particles/fibers. Then, the soluble fractions were left under a flow of air at room temperature to evaporate to constant weight. Note that the degumming conditions significantly affect the performance of UV–Silk resist but have much less effect on UV–LC resist.

*Synthesis of Photosensitive l‐Fibroin (UV–LC)*: The l‐fibroin photoresist was synthesized via chemical conjugation between l‐fibroin and photocrosslinkers (2‐Isocyanatoethyl methacrylate, IEM) in an anhydrous environment. l‐fibroin was suspended at 1% (w/v) in a solution of 1 m LiCl/Dimethyl Sulfoxide (DMSO) and stirred at 65 °C in a dry N_2_ atmosphere for 40 min. Immediately after, the IEM was added at a stoichiometric equivalent to reactive hydroxyl‐containing amino acids and reacted for 5 h at 65 °C. The product was precipitated out, centrifuged, washed and freeze‐dried, sequentially. For comparison, silk fibroins under 10, 30, 60, 90 min degumming time, as well as SilkHTP were prepared and synthesized to be photoreactive following the similar procedure in the Supporting information.

*Photolithography*: Microscale patterns of fibroin were fabricated using photolithography. A solution of 2% (w/v) photoresist was prepared using HFIP (Sigma Aldrich, St. Louis, MO) as a solvent and 0.5% (w/v) of Irgacure 2959 (Sigma Aldrich, St. Louis, MO) as a photoinitiator. The principle of solvent selection is discussed in the Supporting Information. The photoresist solution was then cast at 0.5 mg per substrate. Contact photolithography was conducted using a photomask under a UV exposure (Lumen Dynamics OmniCure 1000, 320–500 nm filter). The unexposed and uncrosslinked protein photoresist was developed using deionized water (18.2 mΩ cm) for 2 h followed by copious rinsing with deionized water and ethanol. Substrates with the developed protein patterns were then dried in a gentle stream of dry N_2_.

*FTIR*: To confirm the methacrylate conjugation, FTIR was conducted on unmodified silk fibroin proteins and l‐fibroin film using a Nicolet iS10 FTIR spectrometer. Cast films (5.0 mg) were analyzed in ATR mode using a Ge ATR crystal, and data was collected between 4000 and 1000 cm^−1^, for 32 scans at a resolution of 1 cm^−1^.

*s‐SNOM*: A commercially available scattering‐type s‐SNOM (Neaspec GmbH, Germany) was utilized with a QCL IR laser (MIRCat, Daylight solutions Inc., USA) tunable between 1495 and 1790 cm^−1^. During instrument operation, the laser was attenuated to ∼10 mW such that the detector yields a nominal signal of 1.5 V. The AFM was operated in tapping mode with 65 nm tapping amplitude. Gold‐coated AFM tips with about 250 kHz resonance (Tap300G‐B‐G, budgetsensors.com) were used to achieve decent IR near‐field signal. The IR near‐field signal was detected simultaneously with AFM signals, using pseudoheterodyne technique and a lock‐in amplifier. The lockin frequency was set at the second or third harmonics of the tip tapping frequency which yield background‐free near‐field amplitude and phase information with a spatial resolution down to 10 nm. The image was scanned at 3.3 ms per pixel for a 500 × 500 pixel sized image.^51^

*Bioactivity Test of Silk Protein Photoresist*: HRP (Sigma Aldrich) was mixed with aqueous UV–LC solution to a final concentration of 0.2 unit mL^−1^. The HRP‐containing UV–LC solution was spin coated onto a quartz substrate to a thickness of 100 nm, and floor UV exposure treatment using the previously described method. The remaining resist was exposed to 3,3′,5,5′‐TMB solution (Sigma Aldrich) to test the activity of the HRP stabilized within the UV–LC resist.

*Etching Process*: All etching process was carried out in a March anisotropic reactive ion etch plasma system. The reactive ion etcher was capable of etching oxides to remove organic contamination from sample surfaces, as well as, tetrafluoromethane (CF_4_) to etch silicon and patterned silk proteins on the silicon substrate. The unit was outfitted with 4 mass flow controllers for reproducible gas flow. O_2_ gas was dissociated in a microwave discharge (100 W) 20 cm upstream of the etching chamber for 10 min to remove the contamination from glass surface. The flow rate of the gas (89.0 L min^−1^) was controlled using mass flow controller. After the cover glass was cleaned, a series of patterned silk protein samples were placed on the holder in the device. CF_4_ gas was dissociated in the microwave discharge of 50 W with flow rate of 48.0 L min^−1^ for 60 s to etch the samples. The etch rate of samples was obtained by measuring thickness change rate via AFM.

*Cell Culture*: Human fetal neural stem cells were subcultured when they were over 90% confluent. Cells were plated in a medium consisting of Dulbecco's Modified Eagle Medium (DMEM)/F12 (GIBCO), 10% fetal bovine serum (GIBCO), penicillin/streptomycin (GIBCO), and 3.5 × 10^−3^ m glucose (Sigma), supplemented with B27 (GIBCO), 10 ng mL^−1^ EGF (Invitrogen), and 10 ng mL^−1^ FGF2 (Invitrogen). Cells were maintained at 37 °C in humidified air with 5% CO_2_.

*Immunofluorescence Assay*: For cell culture staining, the cultures were fixed in 4% Polyformaldehyde (PFA) in Phosphate Buffer Saline (PBS) for 15 min at room temperature. Cells were first washed three times by PBS and then pretreated in 0.1% Triton X‐100 in PBS for 15 min, followed by incubation in 4% normal donkey serum, and 0.1%Triton X‐100 in PBS for 30 min. Primary antibodies (first ab source rabbit, Nestin abcom) were incubated with cultures overnight at 4 °C in 2% normal donkey serum, and 0.1% Triton X‐100 in PBS. After additional washing in PBS, the samples were incubated with appropriate secondary antibodies (second ab donkey anti rabbit 488 flour) conjugated to Alexa Fluor 488 Alexa Fluor.

## Conflict of Interest

The authors declare no conflict of interest.

## Supporting information

Supplementary
