# Supplementary material for: Precise Protein Photolithography (P3): High Performance Biopatterning Using Silk Fibroin Light Chain as the Resist
Source: Adv Sci (Weinh). 2017 Jul 6;4(9):1700191. doi: 10.1002/advs.201700191 (PMC5604371; doi:10.1002/advs.201700191)
Supplement: Supplementary file 1 — Supplementary [file ADVS-4-na-s001.pdf]

Copyright WILEY-VCH Verlag GmbH & Co. KGaA, 69469 Weinheim, Germany, 2017.

ADVANCED SCIENCE Open Access

Supporting Information

for *Adv. Sci.,* DOI: 10.1002/advs.201700191

Precise Protein Photolithography (P^3): High Performance Biopatterning Using Silk Fibroin Light Chain as the Resist

Wanpeng Liu, Zhitao Zhou, Shaoqing Zhang, Zhifeng Shi, Justin Tabarini, Woonsoo Lee, Yeshun Zhang, S. N. Gilbert Corder, Xinxin Li, Fei Dong, Liang Cheng, Mengkun Liu, David L. Kaplan, Fiorenzo G. Omenetto, Guozheng Zhang, Ying Mao,* and Tiger H. Tao**

Copyright WILEY-VCH Verlag GmbH & Co. KGaA, 69469 Weinheim, Germany, 2017.

Supporting Information

DOI: 10.1002/advs.201700191

Precise Protein Photolithography (P^3): High Performance Biopatterning Using Silk Fibroin Light Chain as the Resist

Wanpeng Liu†, Zhitao Zhou†, Shaoqing Zhang†, Zhifeng Shi, Justin Tabarini, Woonsoo Lee, Yeshun Zhang, S. N. Gilbert Corder, Xinxin Li, Fei Dong, Liang Chen, Mengkun Liu, David L. Kaplan, Fiorenzo G. Omenetto, Guozheng Zhang, Ying Mao* and Tiger H. Tao**

Fabrication of silk fibroin photoresists

Silk fibroin proteins were prepared using the established purification protocols [1]. *B. mori* cocoons were boiled for 30 min in aqueous 0.02 M Na_2CO_3 (Sigma-Aldrich, USA) and then rinsed for 3 × 30 min in distilled water to remove the Na_2CO_3 and sericin. The degummed cocoons were allowed to dry for more than 12 h and then subsequently dissolved in 9.3 M LiBr (Sigma-Aldrich, USA) solution at 60 °C for 4 h. The solution was dialysed for 2 days in distilled water using Slide-a-Lyzer dialysis cassettes (MWCO 3,500, Pierce, USA). The solution was centrifuged for 2 × 20 min at 18,000 r.p.m. The concentration was determined by measuring a volume of solution and the final dried weight.

Silk fibroin photoresist was synthesized via chemical conjugation between silk fibroin protein and the reactive isocyanate group of 2-isocyanatoethyl methacrylate (IEM) in an anhydrous solvent system to prevent premature isocyanate reaction [2]. Lithium chloride, silk fibroin protein, and all glassware were thoroughly dried prior to use. Silk fibroin protein was suspended at 1% (w/v) in a solution of 1M LiCl/DMSO and stirred at 65 °C in a dry N_2 atmosphere for 40 minutes, at which point no solids remained suspended in solution. Immediately after, the reagent IEM was added at a stoichiometric equivalence to reactive hydroxyl-containing amino acids. The reaction was allowed to proceed to completion for 5 hours at 60 °C. The product after the reaction was precipitated out into cold ethanol, and centrifuged. The solid product was then washed with a mixture of cold ethanol/acetone and

centrifuged three times. Finally, lyophilization for 48 hours yielded a pure white powder.

Solubility of silk fibroin protein

Solubility was initially investigated to facilitate analysis of the fabrication process. Native silk fibroin protein displays limited solubility when extracted from silk fiber, owing to its secondary structural content. [3] But, with the increase of degumming time, the silk fibroin proteins display enhanced solubility (**Figure S1**) due to the shorter protein chain length. In addition, the silk fibroin protein preliminary dissolution in salt solution, a process which 'activates' the fibroin. [4] Alcohols are known to follow a trend of protein solubilization: HFIP > trifluoroethanol (TFE) > isopropanol > ethanol > methanol, where the fluorinated alcohols HFIP and TFE are specifically known to enhance solubility thorough stabilization of the α-helical conformation. [5] Furthermore, the solubility of silk fibroin protein in non-aqueous solvents DMSO, N,N-dimethylacetamide (DMAc), and N,N- dimethylformamide (DMF) is negligible without the use of salts to disrupt hydrogen bonding. With the chemical conjugation described, the solubility characteristics of the silk fibroin protein varied significantly since the added groups present hydrophobic alkene and carbonyl groups at modified amino acids which were occupied via the conjugation reaction. Among the above organic solvents, HFIP was the only solvent to provide solubility and was thus used as a carrier for characterization as well as deposition of photoresists. Thus, HFIP was used as the primary carrier of the photoresist for incorporation into later photolithography steps.

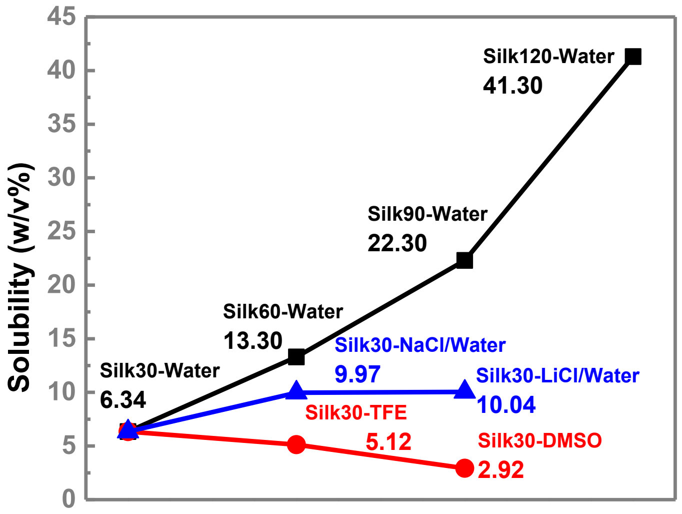

Figure S1. Solubility measurement of silk fibroin proteins under 10, 30, 60, 90 minutes degumming time in different solvents including water, water with salts, TFE and DMSO.

Self-assembly of silk fibroin protein

Silk fibroin protein consists of several polypeptides, thus contributing to a variability in molecular weights ranging from a few tens to a few hundreds of kDa [6]. The protein is characterized by the presence of strongly polar side groups, such as hydroxyl, carboxyl, and amino groups. Silk fibroin protein molecules, thus, follow a diffusion-limited aggregation process (DLA) on surfaces to form nano- and macro-scale architectures with remarkable morphologies and mechanical properties. On drying, silk fibroin protein spontaneously forms branched patterns (**Figure S2**) that are qualitatively and quantitatively similar to the fractal structures formed by a DLA [7] that has been observed in the assembly of a range of materials including colloids, polymer thin films [8], peptides [9, 10], and proteins[11].

To prove that the formation of wrinkled patterns was attributed to the physical phenomenon but not the secondary structure change, the s-SNOM was employed to measure the absorbance and reflection spectrum of the silk film. According to the properties of s-SNOM, if the chromatic difference at 1,631 cm^{-1} (characteristic peak of the secondary structure corresponding to the beta sheet) exists along with the height on the surface, the form of wrinkled patterns is resulted from the secondary structure change. Figure S2(c) indicates the AFM image, and the s-SNOM images of both absorbance and reflection spectrum on the surface of UV-silk30 pattern. No contrast difference in the absorbance and reflection images at 1,635 cm^{-1} was observed, which was attributed to the secondary structure at different regions on the surface were basically the same, and only physical phenomenon existed in the self-assembly procedure depending on properties such as particle size, dispersity and charge.

The underlying substrate is an important factor that may direct the nature of self-assembly of silk fibroin protein. We initially used silicon as the surface in which the surface was treated by IPA solvent. Due to the more hydrophobicity of silicon with the IPA treatment, the solutions tended to dry unevenly, resulting in aggregated clusters and the formation of a film

in many cases (**Figure S2**). We therefore used the substrate without the IPA treatment in the experiments. The relevant hydrophilic and atomically flat nature of the substrate permitted us to spread the solution and to dry it with smaller concentration gradients, yielding high-resolution AFM images. In addition, the lyophilization process did not change the behavior of silk fibroin protein.

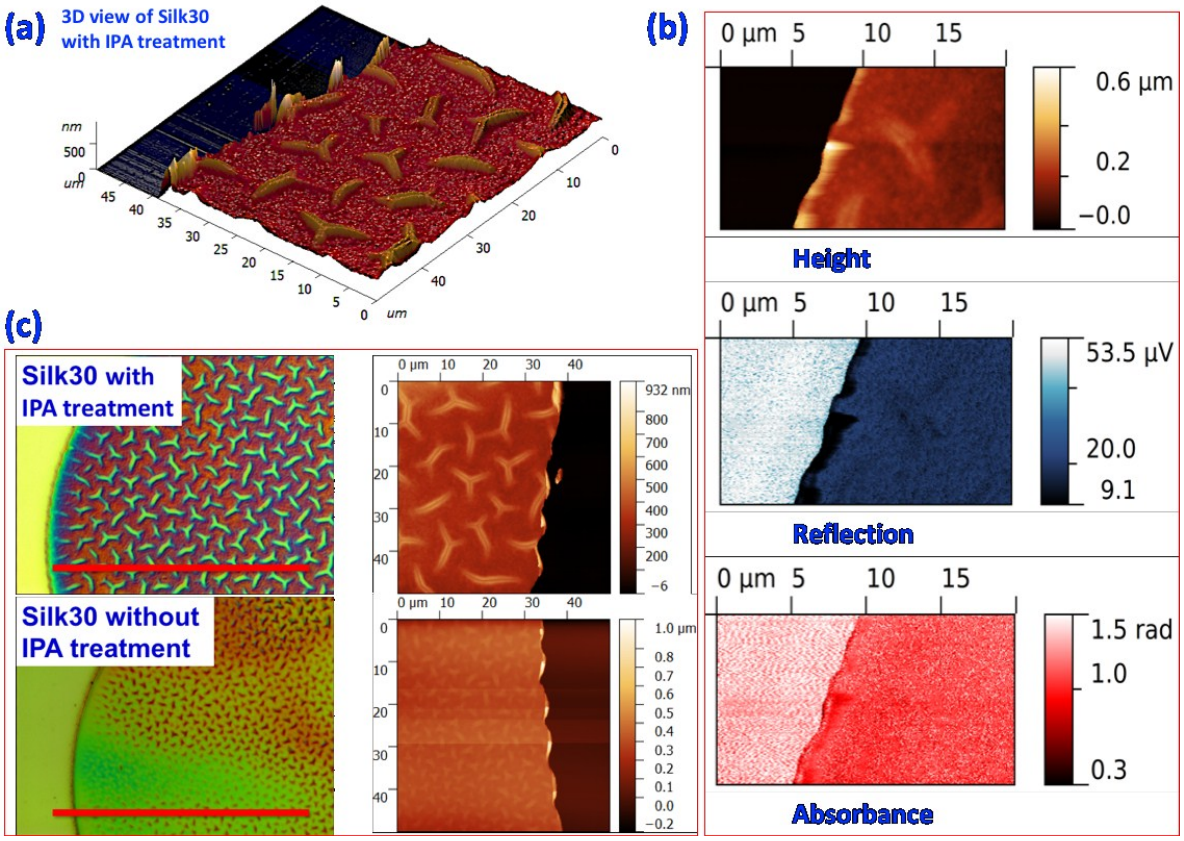

Figure S2. (a) AFM image of 3D view of the surface of silk fibroin protein under 30 minutes degumming time; (b) AFM image and SNOM images of both absorbance and reflection spectrum of the surface of silk fibroin protein under 30 minutes degumming time; (c) Microscope and AFM images of the silk fibroin protein under 30 minutes degumming time to compare the influence of underlying substrate.

Measurements of Resolution

The resolution characterization has been carried out using a Siemens Stars method which is widely used in imaging quality test of optical instruments, printers and displays (https://en.wikipedia.org/wiki/Siemens_star). The method provides information about the resolution at different positions in the image. It is stable and leads to reliable results especially in the case of sharpening and compression algorithms. Due to the self-assembly phenomenon of silk proteins on the silicon substrate, when the pattern dimension decreases to a certain size, the pattern cannot reflect the precise shape of mask. In this case, by detecting the lines in the center of the Siemens Star, we observed swallow-tailed phenomenon of silk resists (the area between two red circles in Figure S3) which was attributed to the self-assembly properties of silk proteins. By measuring and calculating the size of outer circle, we can obtain the resolution of the patterns. The surface roughness has been characterized using Atomic Force Microscopy.

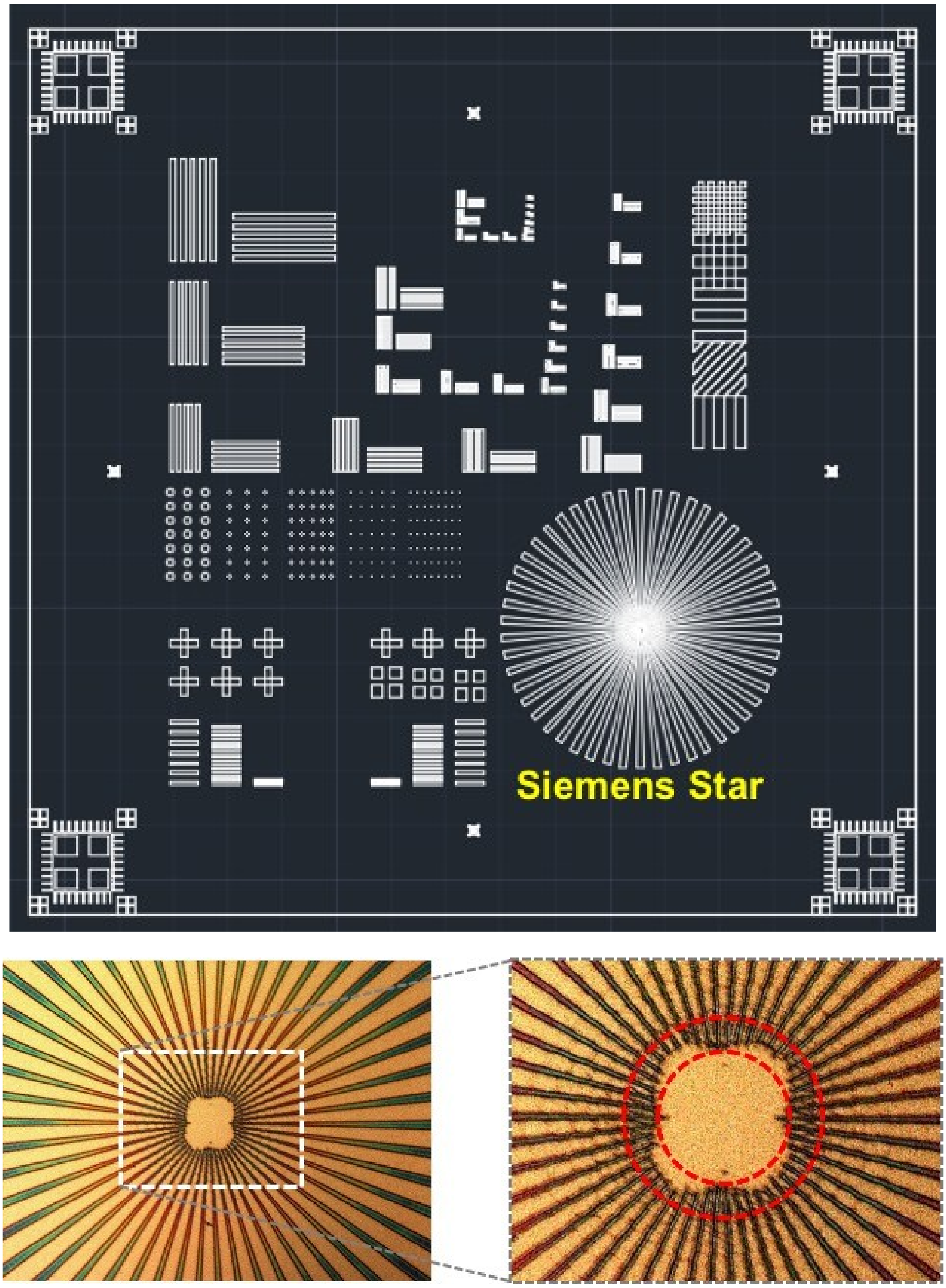

Figure S3. Resolution characterization of silk protein micropatterns by using a Siemens Star method.

Variation of Resolution with Developing Time

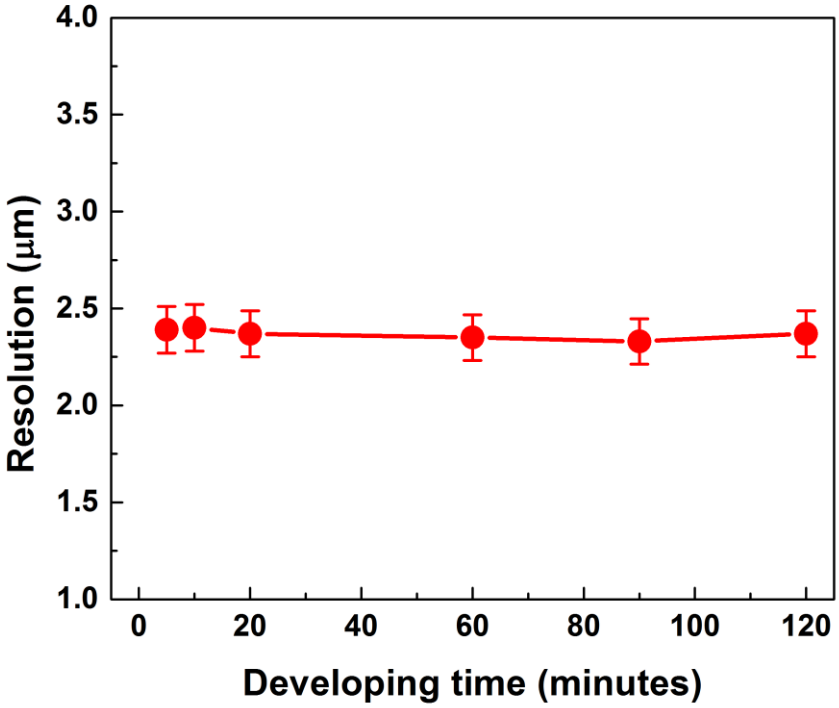

Figure S4. Quantitative analysis of resolution of micropatterns fabricated with developing time.

SDS-PAGE analysis

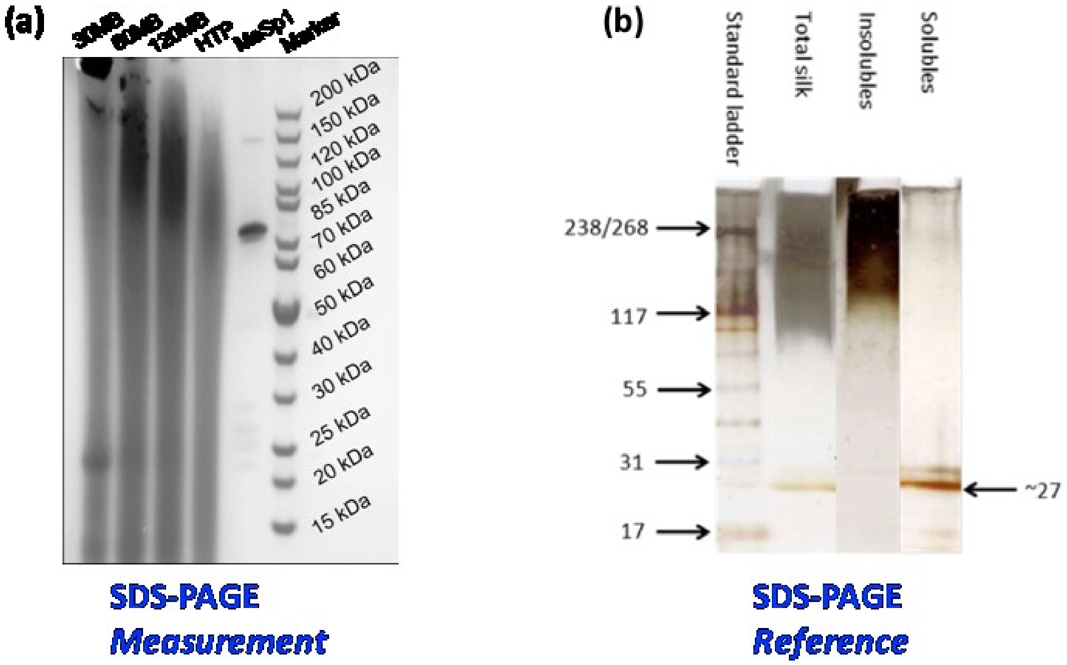

Figure S5. (a) SDS-PAGE analysis of the silk fibroin protein with different degumming time, MaSp1: recombinant spider silk protein; (b) SDS-PAGE analysis of the silk fibroin protein and L-Fibroin from reference [12].

Fourier transform infrared spectroscopy (FTIR)

Fourier transform infrared spectroscopy (FTIR) was employed on unmodified silk fibroin proteins film to confirm the presence of the methacrylate moiety. Cast films (5.0 mg) were analyzed in attenuated total reflectance (ATR) mode using a Ge ATR crystal, and data was collected between 4000 – 1000 cm^{-1}, for 32 scans at a resolution of 1 cm^{-1}. Here, comparison of spectra to that of the IEM, demonstrates functionalization of IEM to different silk fibroin protein under 10, 30, 60, 90 minutes degumming time.

Native silk fibroin under 10, 30, 60, 90 minutes degumming time shows intrinsic Amide I/II/III signals (**Figure S6 (a)**), which are simultaneously observed for the silk fibroin protein photoresist. The IEM reagent (**Figure S6 (b)**) shows stretching vibrations at 1720 cm^{-1}, representing carbonyl ester C=O, and 1640 cm^{-1}, assigned to vinyl C=C (which confers photoreactivity), and an additional intense peak is present at 1160 cm^{-1}, resulting from CH_3 rocking vibrations[13]. The silk fibroin protein photoresist under 30 minutes degumming time also shows peaks representing an ester carbonyl, terminal C=C stretching vibrations as broad shoulder peaks of Amide I, and CH_3 rocking vibrations, all provided by the methacrylate group. The absence of this isocyanate stretching vibration from the product indicates the absence of residual isocyanate. The clear observation of characteristic IEM groups in the product provides evidence to support the successful grafting of IEM onto the silk fibroin protein. In addition, no obvious enhancement of methacrylate group peak with an increase of IEM ratio during the fabrication of silk fibroin photoresists (**Figure S6 (b)**). The secondary structural content of the polypeptide backbone remains relatively unchanged with the methacrylate bioconjugation reaction [14].

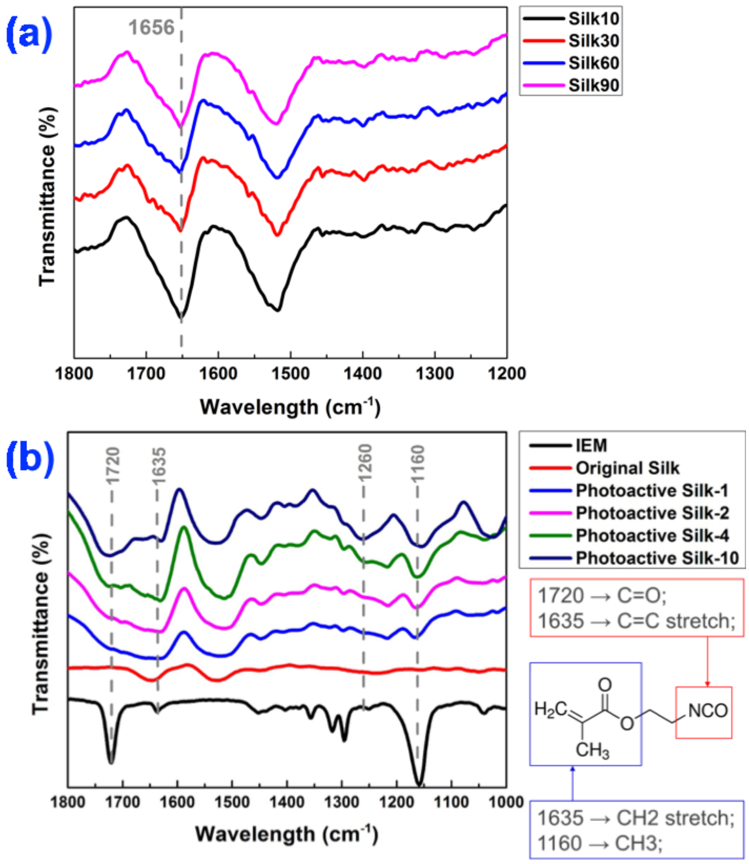

Figure S6. FTIR spectroscopy on the silk fibroin proteins confirms the presence of both amide I/II/III peaks from native silk fibroin proteins, in addition to stretching vibrations at 1720 cm^{-1} (ester carbonyl C=O) and 1630 cm^{-1} (vinyl C=C), and vibrations at 1160 cm^{-1} (CH_3) from the methacrylate substituent.

s-SNOM

s-SNOM set-up employing a tunable single line IR quantum cascade laser (1,450 to 1,750 cm^{-1}) for tip illumination (**Figure S7**). During instrument operation, the light backscattered from the tip is collected and analyzed with a Michelson interferometer operating in pseudo-heterodyne mode. The laser was attenuated to ~10 mW such that the detector yields a nominal signal of 1.5 V. The AFM was operated in tapping mode with 65 nm tapping. Gold-coated AFM tips with about 250 kHz resonance (Tap300G-B-G, budgetsensors.com) were used to optimize the IR near-field signal. The IR signal was detected simultaneously with AFM signals. The IR signal used for analysis in this work was measured by a pseudo-heterodyne technique and a lock-in amplifier. Such amplifier was set at the second and third harmonics of the tapping frequency which provides both reflection and absorption that are (mostly) free of background. Both amplitude and phase information is collected. The near-field interaction leads to a phase spectrum that resembles a familiar molecular absorbance band, while the near-field amplitude spectrum acquires a dispersive line shape similar to a far-field reflectivity spectrum [15].

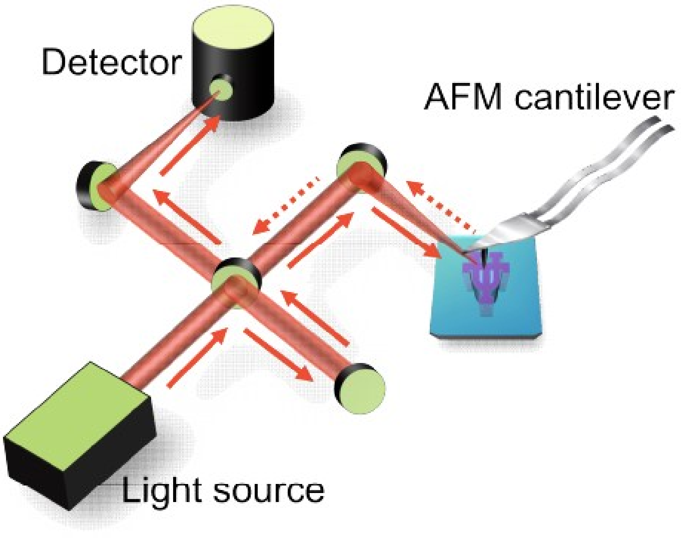

Figure S7. Experimental set-up of s-SNOM equipment.

Young's Modulus measurement

The Young's Modulus is measured by the Force-Distance Spectroscopy module of a commercial AFM (NT-MDT, Russia). The AFM records the applied Z-movement of the cantilever and the real deflection of the tip. The Young's modulus can then be calculated (given a simple model of the AFM tip). For all the measurement, a single ARROW-NCPT tip (NanoWorld, Switzerland) is used and the tip radius is assumed to be 25 nm according to the specification.

Patterns of silk fibroin protein with contact photolithography

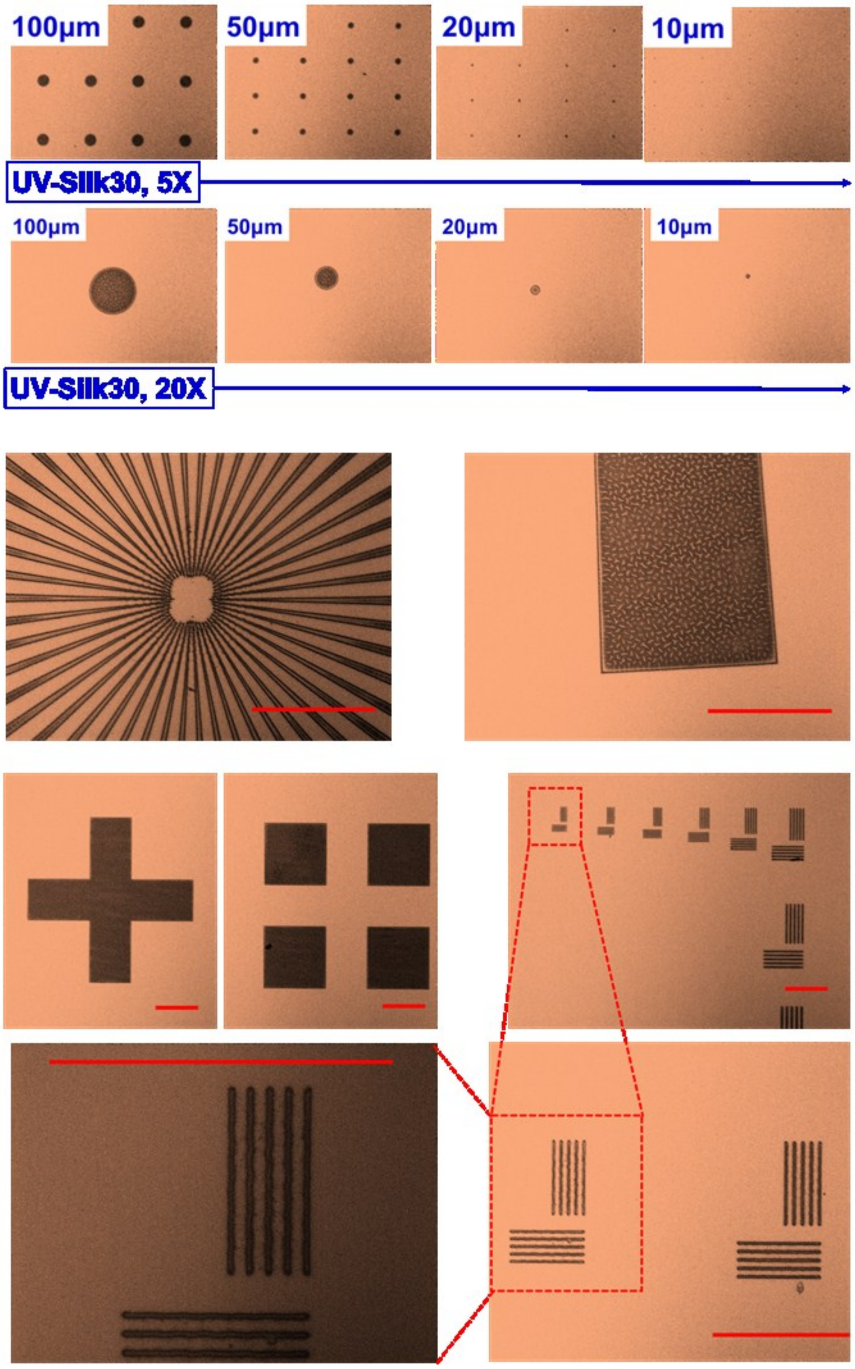

Figure S8. Microscope images of the patterns of Silk fibroin protein using the contact photolithography procedure. All scale bars in the images are 200 μm.

Patterns of L-fibroin protein with contact photolithography

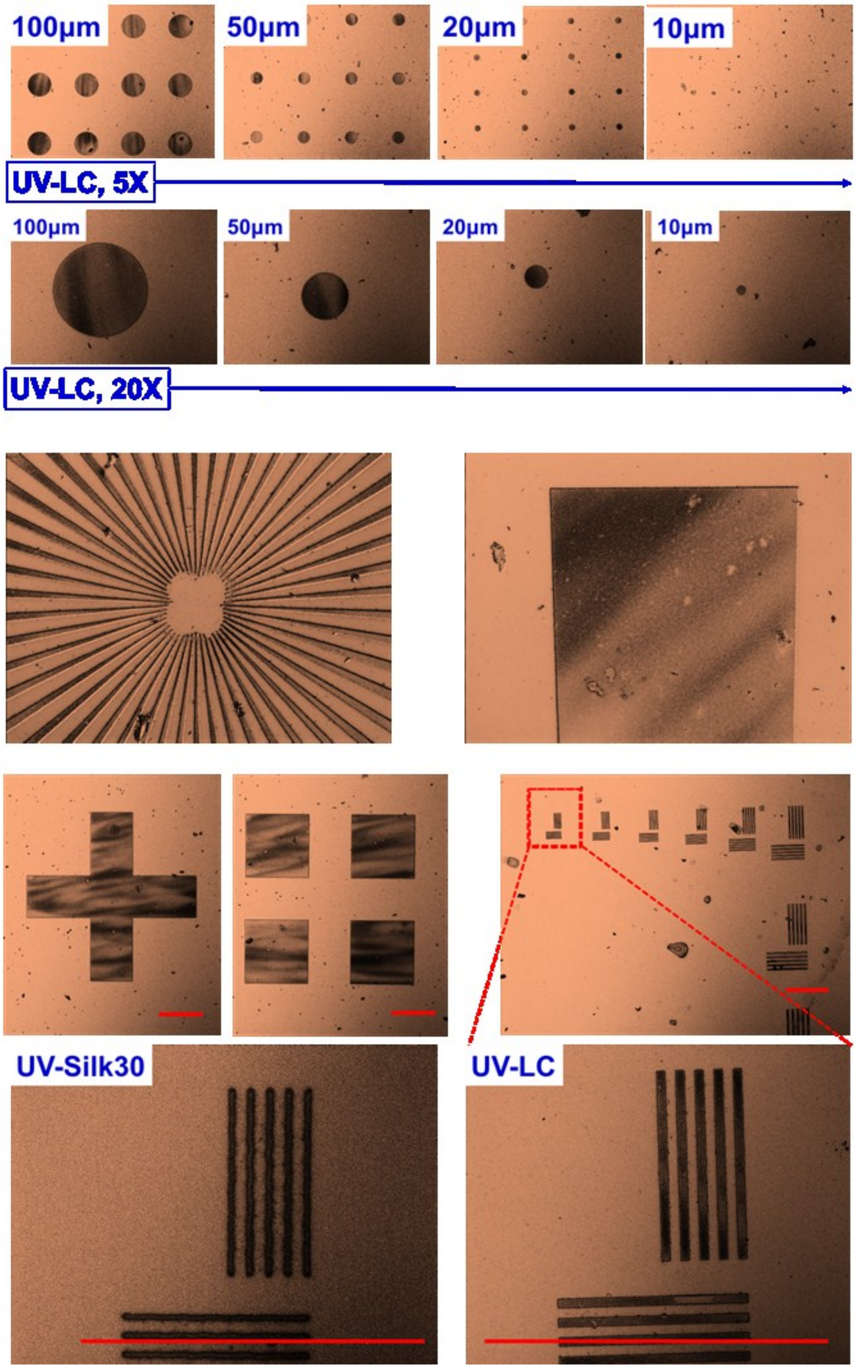

Figure S9. Microscope images of the patterns of L-fibroin protein using the contact photolithography procedure. All scale bars in the images are 200 μm.

References

[1] D. N. Rockwood, et al. *Nature Protocols* **2011**, 6, 1612

[2] H. Teramoto, K.-i. Nakajima, C. Takabayashi, *Biomacromolecules* **2004**, 5, 1392

[3] H. Teramoto, M. Miyazawa, *Biomacromolecules* **2005**, 6, 2049

[4] E. Sashina, A. Bochek, N. Novoselov, D. Kirichenko, *Russian Journal of Applied Chemistry* **2006**, 79, 869

[5] N. Hirota, K. Mizuno, Y. Goto, *Protein Science* **1997**, 6, 416

[6] S. C. Kundu, B. C. Dash, R. Dash and D. L. Kaplan, *Progress in Polymer Science* **2008**, 33, 998

[7] T. A. Witten and L. M. Sander, *Physical Review Letters* **1981**, 47, 1400

[8] G. Reiter, I. Botiz, L. Graveleau, N. Grozev, K. Albrecht, A. Mourran, M. Moller, *Berlin Heidelberg* **2007**, 714, 179

[9] A. Lomander, W. M. Hwang and S. G. Zhang, *Nano Letter* **2005**, 5, 1255

[10] R. Sneer, M. J. Weygand, K. Kjaer, D. A. Tirrell, H. Rapaport, *ChemPhysChem* **2004**, 5, 747

[11] M. M. Murr and D. E. Morse, *Proceedings of the National Academy of Sciences of the United States of America* **2005**, 102, 11657

[12] M. S. Zafar, D. J. Belton, B. Hanby, D. L. Kaplan, C. C. Perry, *Biomacromolecules* **2015**, 16, 606

[13] B. Kaczmarczyk, B. Morejko-Buz, A. Stolarzewicz, *Fresenius' Journal of Analytical Chemistry* **2001**, 370, 899

[14] N. E. Kurland, T. Dey, S. C. Kundu, V. K. Yadavalli, *Advanced Materials* **2013**, 25, 6207

[15] A. A. Govyadinov, I. Amenabar, F. Huth, P.S. Carney, R. Hillenbrand, *The Journal of Physical Chemistry Letters* **2013**, 4, 1526
